# Distribution of five clinically important neuroglial proteins in the human brain

**DOI:** 10.1186/s13041-022-00935-6

**Published:** 2022-06-29

**Authors:** Karl Sjölin, Kim Kultima, Anders Larsson, Eva Freyhult, Christina Zjukovskaja, Kanar Alkass, Joachim Burman

**Affiliations:** 1 Department of Medical Sciences, Neurology, Uppsala University Hospital, 751 85 Uppsala, Sweden; 2 Department of Medical Sciences, Clinical Chemistry, Uppsala University Hospital, 751 85 Uppsala, Sweden; 3 Department of Cell and Molecular Biology, Uppsala University, 751 24 Uppsala, Sweden; 4 Forensic Medicine Laboratory, Department of Oncology-Pathology, Karolinska Institute, 171 77 Stockholm, Sweden; 5 Department of Clinical Neurophysiology, Neurosurgery and Neurology, Uppsala University Hospital, 751 85 Uppsala, Sweden

**Keywords:** Glial fibrillary acidic protein, Myelin basic proteins, Neurofilament proteins, Tau, Hydrolase, ubiquitin carboxy terminal, Central nervous system, Brain, Biomarkers, Atlases as topic

## Abstract

Glial fibrillary acidic protein (GFAP), myelin basic protein (MBP), neurofilament light chain (NFL), tau and ubiquitin carboxy-terminal hydrolase L1 (UCHL1) are five neuroglial proteins that are used as CSF or blood biomarkers of tissue damage in the nervous system. There is incomplete knowledge of how the concentration of these proteins differs between anatomical regions in the CNS as previous studies have focused on gene expression or non-quantitative protein analyses, limiting the interpretability of these biomarkers. The purpose of this study was to create a map of the tissue content of these proteins in different regions of the CNS. The concentrations of the investigated proteins were determined with ELISA in post mortem tissue homogenates from 17 selected anatomical regions in the CNS from ten deceased donors aged 24 to 50 years. When appropriate, the protein concentrations were adjusted for post-mortem interval. In total, 168 tissue samples were analysed. There was a substantial variation in the concentrations of GFAP, MBP, NFL, tau and UCHL1 between different CNS regions. Highly myelinated areas of the CNS had tenfold higher MBP concentration than cerebral cortex, whereas tau showed an inverse pattern. GFAP, NFL and tau displayed an anteroposterior gradient in cerebral white matter. The cerebellum had low concentrations of all the investigated proteins. In conclusion, the tissue concentrations of GFAP, MBP, NFL, tau and UCHL1 were determined throughout the CNS. This information can be used as a reference when interpreting circulating levels of these biomarkers in relation to the extent and localisation of CNS-damaging processes.

**Supplementary Information:**

The online version contains supplementary material available at 10.1186/s13041-022-00935-6.

## Introduction

When neurons and glia are injured, intracellular neuroglial proteins are released into body fluids. Measurement of such proteins in CSF or blood can be used to detect and assess injury to the nervous system in neurological diseases and trauma, e.g. multiple sclerosis, amyotrophic lateral sclerosis, stroke and traumatic brain injury [1–3]. Many neuroglial proteins are unique to one cell type. Measurement of these proteins therefore provides information about which type of cell that is involved in a disease process of interest. The neuroglial proteins glial fibrillary acidic protein (GFAP), myelin basic protein (MBP), neurofilament light chain (NFL), tau and ubiquitin carboxy-terminal hydrolase L1 (UCHL1) are to a large extent selectively expressed in the nervous system and corresponds to different CNS cell types that are differentially distributed in the brain and spinal cord [4, 5]. Recent technological advances allows for sensitive analysis of neuroglial proteins in peripheral blood [6]. However, there is incomplete understanding of how the concentration of neuroglial proteins differs between anatomical regions in the CNS, putting a limit on how we can interpret circulating levels of these proteins in relation to localisation and extent of CNS-damaging disease. The aim of this study was to measure the concentration of GFAP, MBP, NFL, tau and UCHL1 in selected anatomical regions of the human brain and spinal cord.

## Materials and methods

### Tissue collection

Post-mortem tissue was procured from 10 donors by KI Donatum, a core facility at Karolinska Institutet providing post-mortem tissue to researchers through an established donation program (https://ki.se/en/onkpat/ki-donatum). Potential donors were excluded if they had a history of neurological disease. No formal criterion of maximum post-mortem interval was used. Warm time was defined as time from death to start of cold storage at the morgue. Cold time was defined as time from start of cold storage to tissue collection. Tissue from 17 selected anatomical regions in the CNS were obtained from every donor and frozen at − 35 °C in isopentane, and then stored at − 80 °C until analysis.

### Protein extraction and analysis

Tissue samples were thawed and homogenised on ice with a hand-held sonicator in a lysing buffer with protease inhibitors (N-PER™ Neuronal Protein Extraction Reagent, with the addition of Halt™ Protease Inhibitor Cocktail, Thermo Scientific™). After centrifugation at 10.000*g* at 4 °C for 10 min, the supernatant was collected, aliquoted and stored at − 80 °C. The total protein concentration in the supernatant was quantified (Pierce™ BCA Protein Assay Kit, Thermo Scientific™) to verify adequate protein yield. The concentration of neuroglial proteins in the supernatant was determined by commercially available ELISA kits: NFL (NF-light® ELISA kit, UMAN diagnostics. Catalog no.: 10-7001 CE), GFAP (Human GFAP DuoSet ELISA-kit, R&D Systems™. Catalog no.: DY2594-05), tau (Human tau (total) ELISA kit, Invitrogen™/Thermo Fisher. Catalog no.: KHB0041), UCHL1 (Human UCH-L1/PGP9.5 DuoSet ELISA, R&D Systems™. Catalog no.: DY6007-05), MBP (Human MBP DuoSet ELISA, R&D Systems™. Catalog no.: DY4228-05). The supernatant was diluted 500–1,000,000 times before analysis. Samples were run in duplicates, and samples that were out of range were diluted and re-analysed. In our hands, the pooled coefficient of variation for duplicates was for GFAP 3.1%, geometric mean, for MBP 3.9%, for NFL 2.1% for tau 1.9%, and for UHCL1 1.7%. Test of dilution linearity was performed for two of the kits used (GFAP and UCHL1) (Additional file 1: Figs. S5–S9). A more detailed description of sample handling, preparation and validation is available in the Supplementary Information, Additional file 1.

### Statistical analysis

R version 3.6.3 was used for all statistical analyses. The distributions of all proteins were log-normal and for all statistical tests the protein concentrations were log_2_-transformed. To identify any effect of donor characteristics on protein concentration, linear regression was done separately for each protein and CNS region at a time. Protein concentration (Y) was set as the dependent variable, and warm time, cold time and age were set as independent variables. β are the regression coefficients. Warm and cold time were also log_2_-transformed before analysis, whereas age was not:

$${log}_{2}\left(Y\right)={\beta }_{0}+{\beta }_{warm}*{log}_{2}\left(warmtime\right)+{\beta }_{cold}*{log}_{2}\left(coldtime\right)+{\beta }_{age}*Age$$

Cause of death and sex were not included in the model. To assess any meaningful significance, the P-values from the regression analyses were plotted in histograms, so that every histogram included all CNS regions per protein (Additional file 1: Fig. S1). Thereafter, the distribution of P-values for each protein were assessed visually. If the P-values were evenly distributed the associations were considered non-significant, whereas if the P-values were left-skewed, with a distribution centred around zero they were considered to be significant.

If there was a significant association between a donor characteristic and protein concentration, adjustments were done by subtracting the effect of the donor characteristic and then adding the expected effect of a mean value of the same characteristic. As an example, adjustment for warm time was calculated as follows:

$${log}_{2}\left({Y}_{adjusted}\right)={log}_{2}\left(Y\right)-{\beta }_{warm}*{log}_{2}\left(warmtime\right)+{\beta }_{warm}*mean\left({log}_{2}\left(warmtime\right)\right)$$

The log_2_-transformed protein concentrations were then back transformed. All protein concentrations are presented as geometric means with 95% CI, if not otherwise stated.

## Results

### Donor characteristics

In total, 168 brain samples were collected and analysed. Details of the donor characteristics are presented in Table 1. The mean warm time was 8 h and 49 min, and the mean cold time was 36 h and 59 min. Only one of the donors was female, and the mean age was 42 years (min–max 24–50). Hanging was the most common cause of death (40%), followed by ischemic heart disease (30%).

**Table 1:** Donor characteristics

Donors, no	*n* = 10
Age, mean (min–max), years	42 (24–50)
Female sex, no. (%)	1 (10%)
Cause of death, no. (%)	
Hanging	4 (40%)
Ischemic heart disease	3 (30%)
Unknown	2 (20%)
Pulmonary embolism	1 (10%)
Warm time, mean (min–max), hrs:min	8:49 (2:15–26:09)
Cold time, mean (min–max), hrs:min	36:59 (6:54–48:00)
Tissue samples analysed, no	168^a^

^a^ Samples from hippocampus were missing from two donors

### Distribution of neuroglial proteins

**Fig. 1:**
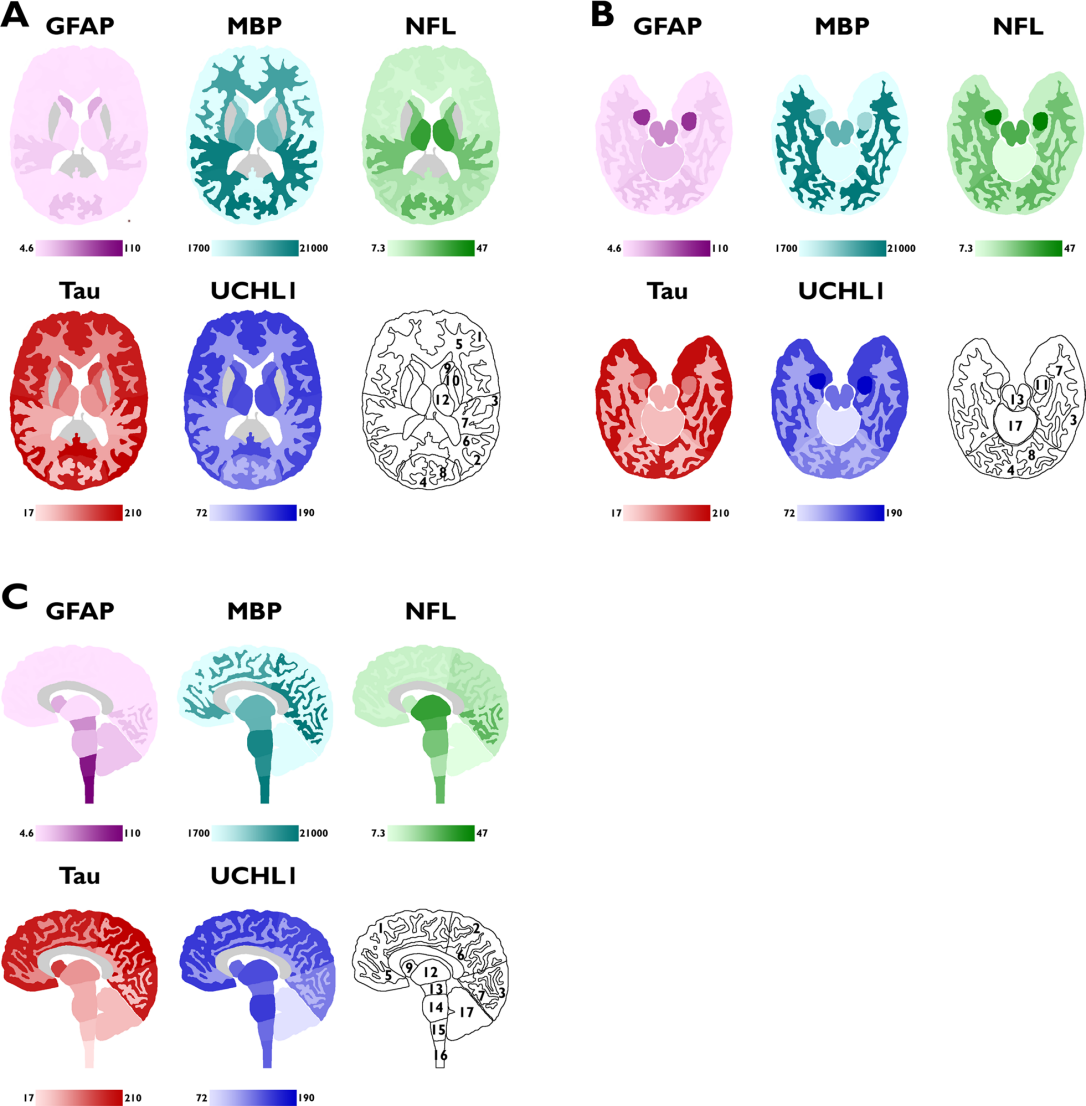
**A** axial cranial, **B** axial caudal, and **C** sagittal sections of the CNS. The bar below every section shows the colour gradient used between the minimum and maximum value for each neuroglial protein. Darker colour represents higher values. Grey areas represent anatomical parts of the brain that were not included in this study. 1 = cerebral frontal cortex, 2 = cerebral parietal cortex, 3 = cerebral temporal cortex, 4 = cerebral occipital cortex, 5 = cerebral frontal white matter, 6 = cerebral parietal white matter, 7 = cerebral temporal white matter, 8 = cerebral occipital white matter, 9 = caudate nucleus, 10 = internal capsule, 11 = hippocampus, 12 = thalamus, 13 = mesencephalon, 14 = pons, 15 = medulla oblongata, 16 = cervical spinal cord, 17 = cerebellum

The concentrations of the neuroglial proteins in selected anatomical regions of the CNS are presented in Table 2. The concentrations are summarised as geometric mean [95% CI] in µg/g (wet weight of CNS tissue), and their distribution is visualised in Fig. 1A–C. The distribution is also visualised in diagrams available in Additional file 1: Fig. S2.

**Table 2:** Concentration of neuroglial proteins in selected CNS regions

CNS region	GFAP (µg/g)	MBP^a^ (µg/g)	NFL^a^ (µg/g)	Tau (µg/g)	UCHL1 (µg/g)	Total protein (mg/g)
Cerebrum						
Cortex						
Frontal	4.8 (1.7–13)	1900 (1500–2500)	12 (8.6–17)	190 (160–220)	160 (110–230)	43 (40–46)
Parietal	5.2 (1.7–16)	2100 (1600–2800)	14 (10–18)	210 (170–270)	150 (110–220)	44 (40–48)
Temporal	5.4 (2.0–15)	1700 (1100–2600)	12 (9.1–17)	200 (180–230)	160 (96–250)	46 (44–49)
Occipital	4.6 (1.3–16)	2300 (1700–3100)	13 (8.9–18)	190 (150–240)	130 (76–210)	42 (39–45)
White matter						
Frontal	5.9 (2.0–17)	16,000 (12,000–20,000)	10 (5.4–19)	94 (73–120)	110 (94–130)	32 (29–36)
Parietal	5.0 (1.6–16)	20,000 (14,000–29,000)	17 (9.9–30)	71 (56–89)	100 (89–120)	31 (28–35)
Temporal	14 (3.7–55)	21,000 (16,000–26,000)	27 (15–50)	64 (47–86)	100 (94–120)	34 (31–38)
Occipital	20 (5.2–79)	21,000 (17,000–27,000)	30 (17–54)	46 (37–59)	95 (79–110)	33 (30–37)
Caudate nucleus	31 (11–82)	3200 (2700–3900)	13 (7.3–23)	160 (150–180)	140 (60–310)	47 (42–52)
Internal capsule	5.9 (1.9–19)	11,000 (7400–16,000)	27 (12–60)	120 (88–170)	150 (94–250)	44 (39–49)
Hippocampus	91 (51–160)	7600 (6000–9500)^b^	47 (18–120)^b^	110 (79–150)	190 (110–310)	49 (45–54)
Diencephalon						
Thalamus	7.6 (2.4–24)	13,000 (7900–21,000)	38 (20–72)	83 (64–110)	150 (72–310)	44 (39–50)
Brainstem						
Mesencephalon	46 (21–98)	13,000 (7100–23,000)	33 (20–56)	62 (45–85)	130 (94–180)	36 (30–42)
Pons	25 (9.0–69)	19,000 (13,000–30,000)	26 (18–38)	62 (49–77)	160 (120–220)	38 (34–43)
Medulla oblongata	100 (61–170)	18,000 (13,000–25,000)	17 (11–25)	42 (39–46)	130 (100–170)	37 (32–42)
Cervical spinal cord	110 (60–220)	21,000 (15,000–30,000)	30 (20–45)	17 (14–22)	140 (120–160)	37 (34–41)
Cerebellum	19 (9.4–38)	2000 (1500–2600)	7.3 (4.8–11)	49 (39–61)	72 (48–110)	45 (42–49)

Values are summarised as geometric mean (95% CI). Units are µg/g of CNS tissue, except for total protein that has the unit mg/g of CNS tissue. All values for GFAP, tau and UCHL1 are unadjusted

^a^Values for MBP and NFL are adjusted for donor warm time, except for hippocampus that did not correlate to warm time and was left unadjusted

^b^Unadjusted value

The P-values from the regression analyses of GFAP, tau and UCHL1 were evenly distributed, indicating that there were no significant association between any donor characteristic and the protein concentrations. Donor warm time (but not donor cold time or age) and concentrations of MBP and NFL had a left skewed distribution of P-values centred around zero. In view of that, concentrations for MBP and NFL were adjusted for warm time in all CNS regions, except hippocampus, that was an outlier and left unadjusted. No other adjustments were made (Additional file 1: Tables S1–S6 and Fig. S1).

#### Total protein

The total protein concentration ranged between 31 [28–35] mg/g in cerebral parietal white matter to 49 [45–54] mg/g in hippocampus. Cerebral white matter, brainstem and cervical spinal cord had lower protein concentration than the other CNS regions.

##### GFAP

The concentration of GFAP was highest in the cervical spinal cord (110 [60–220] µg/g), and lowest in the cerebral cortex, where concentrations ranged from 4.6 [1.3–16] to 5.4 [2.0–15] µg/g. Overall, both cortex and white matter in cerebrum had lower concentrations compared with the brainstem and spinal cord. In cerebral white matter, a tendency of an anteroposterior gradient, with higher concentrations more posteriorly, was noted.

##### MBP

The concentrations of MBP were 10–1000 times higher than the other investigated proteins, depending on anatomical region. The concentration of MBP was highest in cerebral white matter (16,000 [1–12, 12–20] to 21,000 [1–17, 17–27] µg/g) and approximately tenfold to the one in cerebral cortex (1700 [1100–2600] to 2300 [1700–3100] µg/g). Intermediate levels were seen in the hippocampus, internal capsule, thalamus and mesencephalon, with higher concentrations in the rest of the brainstem and cervical spinal cord.

##### NFL

NFL concentrations were higher in the temporal and occipital cerebral white matter (27 [15–50] to 30 [17–54] µg/g) than in the cerebral cortex (12 [8.6–17] to 14 [10–18] µg/g) or frontal and parietal cerebral white matter (10 [5.4–19] and 17 [9.9–30] µg/g), indicating an anteroposterior gradient in the cerebral white matter, with increasing concentration more posteriorly. High concentrations of NFL were also seen in hippocampus, internal capsule, thalamus, mesencephalon, pons, and cervical spinal cord.

#### Tau

The concentrations of tau were highest in the cerebral cortex (190 [160–220] to 210 [170–270] µg/g) and in the caudate nucleus (160 [150–180] µg/g), whereas the cervical spinal cord had the lowest concentration, 17 [14–22] µg/g. In the cerebral white matter, brainstem, and spinal cord, concentrations ranged from 120 [88–170] µg/g in the internal capsule to 17 [14–22] µg/g in the cervical spinal cord, indicating a craniocaudal and anteroposterior gradient with decreasing concentration more caudally and posteriorly.

#### UCHL1

Throughout the cerebrum, the concentration of UCHL1 was higher in cortex (130 [76–210] to 160 [96–250] µg/g) than in white matter (95 [79–110] to 110 [94–130] µg/g). The highest concentration was seen in hippocampus (190 [110–310] µg/g) and the lowest in cerebellum (72 [48–110] µg/g).

## Discussion

In this study, the concentrations of GFAP, MBP, NFL, tau and UCHL1 were determined in 17 anatomical regions of the CNS. Our findings indicate a substantial regional variation in the concentration of the investigated proteins. The concentration of GFAP was twenty times higher in the medulla oblongata and cervical spinal cord, compared with cerebral cortex. The concentration of MBP in highly myelinated areas of CNS (cerebral white matter, pons, medulla oblongata and cervical spinal cord) was tenfold higher than in cerebral cortex. In contrast, tau had an inverse relationship between cerebral cortex and white matter, with higher concentrations in all parts of cerebral cortex compared with cerebral white matter. GFAP, NFL and tau displayed an anteroposterior gradient in the cerebral white matter, with higher concentrations more posteriorly for GFAP and NFL, and the opposite for tau. In the cerebrum, UCHL1 concentration was slightly higher in sections of grey matter (cerebral cortex, caudate nucleus, and hippocampus) than in white matter, but the concentration in the internal capsule, thalamus, brainstem and cervical spinal cord did not differ from the cerebral cortex. Among the studied regions, the cerebellum stands out, with generally low concentrations of all the investigated proteins.

The warm time affected the levels of both MBP and NFL; higher concentrations were associated with increased duration of warm time (Additional file 1: Figs. S3, S4). This could be due to post-mortem cell degradation releasing proteins from cell structures. In a study on post-mortem changes in rat brain, most changes in protein levels occurred after 24 h, and some protein levels increased post-mortem, but not NFL, which in that study decreased [7]. Another study on post-mortem concentration of NFL in human frontal cortex could not confirm any correlation with post-mortem interval [8]. Contrary to our result, MBP concentration decreased with increasing post-mortem interval in a study on vascular white matter changes and dementia [9]. The lack of association between protein concentrations and duration of cold time should be expected, since low temperature retards the post-mortem degradation processes.

To our knowledge, there is no previous study that has systematically quantitated the concentration and distribution of these proteins throughout the CNS. Previous studies on these proteins using ELISA on CNS tissue homogenates are scarce and have focused on a single or a few CNS regions. Petzold et al. reported a GFAP concentration of 1.7 (1.1–5.9) (median, range) µg/mg of total protein in cerebral white matter and 0.8 (0.5–1.2) (median, range) µg/mg of total protein in cerebral cortex from five deceased control patients in a study on multiple sclerosis [10]. In another study, GFAP concentration of 0.8 (0.6–1.1) (median, IQR) µg/mg of total protein in non-lesional white matter in 12 patients with multiple sclerosis was reported [11]. These concentrations of GFAP were normalised to total protein concentration, and were similar to the concentrations in our study. In a study on Alzheimer disease, the concentration of tau in cerebral cortex was 115.8 µg/g tissue in a control subject – again, similar to our findings [12]. The total protein yield was on average 4% (w/w), which is similar with a previous study on drug transporter abundance in post-mortem brain tissue from 30 individuals, using the same protein assay [13].

Extensive open access databases describing the human brain proteome are available, such as the Human Protein Atlas (https://www.proteinatlas.org), or the Allen Institute for Brain Science (https://alleninstitute.org) [5, 14]. The information in these databases relies on gene expression data, immunohistochemistry or in situ hybridization. Our results concerning GFAP, MBP, tau and UCHL1 were consistent with gene expression data from the Human Protein Atlas, less so for NFL. For instance, the highest gene expression of GFAP was reported for mesencephalon, medulla oblongata and spinal cord, which is in line with the actual protein distribution we measured in this study. Another example is cerebral cortex, where MBP was reported to have low and tau high gene expression, which is consistent with our results. NFL on the other hand was in the Human Protein Atlas reported to have a relatively low gene expression in the spinal cord, whereas we found relatively high NFL concentrations in the cervical spinal cord. This inconsistency can be explained by the fact that gene expression data does not reliably reflect the concentration of proteins present in the tissue [15]. Immunohistochemistry on the other hand, gives a good spatial view of the protein distribution but lacks quantitative information. Mass spectrometry allows for (semi) quantification of protein abundance in a tissue, and proteomic profiling of some parts of the human brain have been made [16]. However, the relative abundances of our proteins of interest between the seven investigated brain areas included in that study are not presented, making comparisons difficult to perform. In this study we used ELISA, allowing for absolute protein quantification with high specificity, measuring the protein of interest directly through antibody capture. In comparison with gene expression, mass spectrometry and immunohistochemistry, we believe that our approach better reflects the actual amount present in the tissue, and what eventually will get into the blood when a tissue-damaging process affects a certain part of the CNS.

For many years, analysis of cerebrospinal fluid has been a requirement for obtaining a reliable measurement of these proteins. The development of novel highly sensitive analytical assays has made it possible to reliably quantify the concentrations of these neuroglial proteins in blood samples [17, 18]. The simplicity of a blood test increases the clinical usefulness, and analysis of serum NFL is already approaching clinical routine [19, 20]. Knowledge about the actual distribution of these proteins in the CNS provides a foundation for a correct interpretation of circulating levels in relation to size and location of a CNS damage. Several studies have demonstrated a correlation between infarct size and serum or plasma levels of GFAP, MBP, NFL and tau in acute ischemic stroke [21–24]. It is reasonable to assume that a stroke affecting mainly white matter would give another pattern (e.g. high serum levels of MBP but not tau) than a stroke affecting mostly grey matter in cortex or basal ganglia (e.g. low serum levels of MBP but high levels of UCHL1 and tau). There are however no studies specifically investigating such patterns and correlation to stroke location. Correlation with lesion volume is seen in other neurological disorders as well; in a recent study of 197 patients with traumatic brain injury, serum measurements of GFAP, NFL, tau and UCHL1 correlated with lesion volume on MRI [3]. In multiple sclerosis, serum NFL correlates with lesion load, lesion volume and gadolinium enhancing lesions on MRI [25, 26]. Another example is serum measurement of GFAP, that is associated with both disease activity and severity in neuromyelitis optica, an inflammatory disorder targeting astrocytes, leading to attacks of longitudinal myelitis and optic neuritis [27]. These examples will most likely be followed by more in the future.

This study has several limitations. The sample size was small, limited to ten donors. Nevertheless, considering the nature of the study this can be considered a fairly large material. We relied upon protein extractions from post-mortem tissue, where factors like post-mortem interval, cause of death and other characteristics possibly could have affected protein concentration. To remedy this, we searched for associations between donor characteristics and protein concentrations and could only find an association between two of the proteins (MBP and NFL) and warm time, which was then adjusted for. Further, GFAP, MBP, NFL and tau have previously been evaluated as post mortem markers of traumatic brain injury, indicating that these proteins remain stable post-mortem [28, 29]. Cause of death was not included in our model due to the limited number of donors, and there is a possibility that causes of death could influence post-mortem concentrations of neuroglial proteins differently. One alternative approach would have been to use biopsy material obtained at neurosurgery. This was not considered feasible, since it would not permit a systematic collection of tissue from standardised CNS regions and also introduce the possibility of confounding by trauma or disease-specific mechanisms.

Although we used ELISA kits from well-known suppliers, these had not been validated for neuronal tissue homogenates, and there is a risk of matrix interference. To minimize interference, samples were diluted between 500 and 1,000,000 times, with a dilution of 10,000 being the most frequent dilution needed for a correct read-off. A high dilution factor reduces the risk of matrix interference.

In conclusion, in this study we presented how five clinically important neuroglial proteins are distributed in the CNS. There was a substantial variation in the concentration of the investigated proteins and between CNS regions. This information is useful when interpreting circulating levels of these proteins in relation to localisation and extent of a CNS-damaging disease.

## Supplementary Information

**Additional file 1. **Supplementary Methods and Results. Additional description of methods and results, including Tables S1–S7 and Figures S1–S9.

**Additional file 2. **Supplementary Data. Unadjusted data of all ELISA analyses per donor and brain section, including all donor characteristics available for the study.

## Data Availability

All data generated or analysed during this study are included in this published article and its supplementary information files (Additional files 1 and 2).

## Abbreviations

GFAP: Glial fibrillary acidic protein
MBP: Myelin basic protein
NFL: Neurofilament light chain
UCHL1: Ubiquitin carboxy-terminal hydrolase L1
