# Supplementary material for: Distribution of five clinically important neuroglial proteins in the human brain
Source: Mol Brain. 2022 Jun 29;15:52. doi: 10.1186/s13041-022-00935-6 (PMC9241296; doi:10.1186/s13041-022-00935-6)
Supplement: Supplementary file 1 — Additional file 1. Supplementary Methods and Results. Additional description of methods and results, including Tables S1–S7 and Figures S1–S9. [file 13041_2022_935_MOESM1_ESM.docx]

Supplementary Methods and Results

**Content**

[**1. Protein extraction protocol 3**](#_Toc102048063)

[**2. Multiple linear regression analysis 5**](#_Toc102048064)

[**3. Concentration diagrams 13**](#_Toc102048065)

[**4. Association between warm time and concentration of MBP and NFL 14**](#_Toc102048066)

[**5. Validation data 16**](#_Toc102048067)

# 1. Protein extraction protocol

**Material:**

1. Brain samples, provided in 2 ml Eppendorf tubes, stored in -80°C freezer.
2. Lysing buffer, N-PER™ Neuronal Protein Extraction Reagent. Thermo Scientific™. Catalog no.: 87792.
3. Protease inhibitors, Halt™ Protease Inhibitor Cocktail, EDTA-Free (100X). Thermo Scientific™. Catalog no.: 78439
4. Handheld homogenizer (Sonicator)
5. Conical Centrifuge Tubes Falcon™ 50 mL, polypropylene. Falcon Ref no.: 352097.
6. Conical Centrifuge tubes Falcon™ 15 mL, polypropylene. Corning™. Falcon Ref. no.: 352097.
7. Microcentrifuge Tube Eppendorf™ Polypropylene Protein LoBind 1.5 mL. Eppendorf Order no.: 0030108116
8. Cryo preservation tubes, Sarstedt Screw Cap Micro tube 0.5 ml, polypropylene. Ref. no.: 72.730.005
9. Scalpel, spatula, glass plate, methanol, paper swab, ice, dry ice.

**Protocol**

1. Preparation of lysing buffer
   1. 10 µl HALT™ Protease Inhibitor Cocktail is added per 1 ml of N-PER™ (according to the product sheet). Thereafter gentle mix (to avoid formation of bubbles) of the mixture, and store in refrigerator prior to use.
2. Preparation of brain samples
   1. Prepare 1 per sample of 15 ml Falcon conical centrifuge tubes. Every tube is marked and weighed separately and thereafter stored on ice.
   2. Remove the brain samples from the -80°C freezer and put them on dry ice. Thaw 2 samples at a time very shortly on ice (approx. 1 min), just so the tissue loosens from the tube wall.
   3. Place the tissue on a glass plate on ice with a spatula. Cut approx 450-550 mg from the still frozen brain sample. Immediately transfer the remaining part back to the 2 ml Eppendorf tubes and back on dry ice.
   4. Then finely divide the brain tissue with a scalpel and immediately transfer to the 15 ml Falcon™ tubes on ice. The glass plate, scalpel and spatula should be cleaned with a paper swab and thereafter methanol between each sample.
   5. Weigh the 15 ml Falcon™ tubes + brain sample separately and calculate the net weight of brain sample. Keep samples on ice.
   6. Remove or add brain sample if net weight is > 700 mg or < 400 mg for grey substance and < 450 mg for white matter per sample, and calculate net weight again.
   7. Put the remaining brain samples back in -80°C freezer to minimize time on dry ice.
   8. Add lysing buffer (N-PER™ + HALT™ Protease Inhibitor Cocktail) to the brain samples at a ratio of 1 ml/100 mg brain sample. Keep on ice.
   9. Homogenize the brain samples with a hand-held homogenizer (Sonicator) on ice in a fume hood to avoid potential aerosols. Use the following sonicator settings: Amplitude 40, other settings 0. Sonicate in a sequence of 10 seconds x 6 with 10 seconds pause between each 10 seconds of sonication. Wash the Sonicator tip with paper swab and methanol between samples.
   10. Incubate the homogenates on ice for approx. 1 hour.
   11. Transfer the homogenate to Eppendorf™ Protein Lobind microcentrifuge tubes. Usually 3-5 microcentrifuge tubes per homogenate is needed.
   12. Centrifugate the homogenates at 10.000 g in 10 min at 4°C.
   13. Prepare and mark a new set of 15 ml Falcon™ tubes, 1 per brain sample. Put on ice.
   14. Collect the supernatant (protein extract) from the microcentrifuge tubes and put the supernatant for each brain sample together in a 15 ml Falcon™ tube and vortex. Put on ice.
   15. Transfer the protein extract to marked cryo preservation tubes in aliquots of 300 µl, and thereafter immediately put the aliquots in -80°C freezer, for later downstream analyses.

# 2. Multiple linear regression analysis

The results from multiple linear regressions analysis are presented in table S1-S6. Separate analyses were done for each neuroglial protein and CNS region. Numbers in bold represents significance level < 0.05. Due to the limited sample size (n=10) and to identify any biologically meaningful associations, P-values for each analysis where plotted in histograms and assessed visually, where a left skewed distribution centred around zero was considered to be significant (Fig. S1).

**Table S1.** GFAP, multiple linear regression results.

| **Brain section** | **Variable** | **Estimate** | **Std. Error** | **t value** | **Pr(>\|t\|)** |
| --- | --- | --- | --- | --- | --- |
| Frontal lobe cortex | log2(warm time) | -1.136 | 0.629 | -1.806 | 0.121 |
|  | log2(cold time) | -0.697 | 0.721 | -0.967 | 0.371 |
|  | Age | -0.003 | 0.075 | -0.046 | 0.965 |
| Parietal lobe cortex | log2(warm time) | -1.297 | 0.690 | -1.881 | 0.109 |
|  | log2(cold time) | -0.638 | 0.790 | -0.807 | 0.451 |
|  | Age | -0.012 | 0.083 | -0.141 | 0.893 |
| Temporal lobe cortex | log2(warm time) | -1.068 | 0.519 | -2.057 | 0.085 |
|  | log2(cold time) | -0.651 | 0.595 | -1.093 | 0.316 |
|  | Age | 0.036 | 0.062 | 0.579 | 0.584 |
| Occipital lobe cortex | log2(warm time) | -1.627 | 0.610 | -2.667 | **0.037** |
|  | log2(cold time) | -0.817 | 0.699 | -1.169 | 0.287 |
|  | Age | -0.004 | 0.073 | -0.050 | 0.962 |
| Frontal lobe white matter | log2(warm time) | 0.256 | 0.556 | 0.461 | 0.661 |
|  | log2(cold time) | -1.632 | 0.637 | -2.563 | **0.043** |
|  | Age | 0.145 | 0.067 | 2.186 | 0.071 |
| Parietal lobe white matter | log2(warm time) | -0.112 | 0.673 | -0.167 | 0.873 |
|  | log2(cold time) | -1.464 | 0.771 | -1.899 | 0.106 |
|  | Age | 0.123 | 0.081 | 1.526 | 0.178 |
| Temporal lobe white matter | log2(warm time) | -0.540 | 0.914 | -0.590 | 0.577 |
|  | log2(cold time) | -1.442 | 1.048 | -1.376 | 0.218 |
|  | Age | 0.068 | 0.110 | 0.619 | 0.559 |
| Occipital lobe white matter | log2(warm time) | -0.215 | 1.036 | -0.207 | 0.843 |
|  | log2(cold time) | -1.428 | 1.187 | -1.203 | 0.274 |
|  | Age | 0.036 | 0.124 | 0.289 | 0.783 |
| Caudate nucleus | log2(warm time) | -1.286 | 0.450 | -2.859 | **0.029** |
|  | log2(cold time) | -0.290 | 0.515 | -0.563 | 0.594 |
|  | Age | 0.036 | 0.054 | 0.662 | 0.533 |
| Internal capsule | log2(warm time) | -0.985 | 0.724 | -1.361 | 0.222 |
|  | log2(cold time) | -0.250 | 0.830 | -0.301 | 0.774 |
|  | Age | 0.079 | 0.087 | 0.911 | 0.398 |
| Hippocampus | log2(warm time) | -0.331 | 0.396 | -0.836 | 0.450 |
|  | log2(cold time) | -0.277 | 0.424 | -0.654 | 0.549 |
|  | Age | 0.022 | 0.049 | 0.442 | 0.681 |
| Thalamus | log2(warm time) | -0.594 | 0.926 | -0.642 | 0.545 |
|  | log2(cold time) | -0.453 | 1.062 | -0.426 | 0.685 |
|  | Age | 0.024 | 0.111 | 0.217 | 0.836 |
| Mesencephalon | log2(warm time) | -0.594 | 0.565 | -1.052 | 0.333 |
|  | log2(cold time) | -0.538 | 0.648 | -0.831 | 0.438 |
|  | Age | -0.037 | 0.068 | -0.545 | 0.606 |
| Pons | log2(warm time) | -1.173 | 0.753 | -1.557 | 0.170 |
|  | log2(cold time) | 0.295 | 0.863 | 0.342 | 0.744 |
|  | Age | -0.081 | 0.090 | -0.902 | 0.402 |
| Medulla oblongata | log2(warm time) | -0.570 | 0.369 | -1.544 | 0.173 |
|  | log2(cold time) | -0.231 | 0.423 | -0.547 | 0.604 |
|  | Age | -0.024 | 0.044 | -0.549 | 0.603 |
| Cervical spinal cord | log2(warm time) | -0.592 | 0.444 | -1.333 | 0.240 |
|  | log2(cold time) | 2.433 | 3.241 | 0.751 | 0.487 |
|  | Age | -0.110 | 0.108 | -1.021 | 0.354 |
| Cerebellum | log2(warm time) | -0.407 | 0.558 | -0.730 | 0.493 |
|  | log2(cold time) | -0.382 | 0.639 | -0.597 | 0.572 |
|  | Age | -0.011 | 0.067 | -0.170 | 0.871 |

**Table S2.** MBP, multiple linear regression results.

| **Brain section** | **Variable** | **Estimate** | **Std. Error** | **t value** | **Pr(>\|t\|)** |
| --- | --- | --- | --- | --- | --- |
| Frontal lobe cortex | log2(warm time) | 0.189 | 0.161 | 1.177 | 0.284 |
|  | log2(cold time) | 0.445 | 0.184 | 2.418 | 0.052 |
|  | Age | 0.003 | 0.019 | 0.156 | 0.881 |
| Parietal lobe cortex | log2(warm time) | 0.557 | 0.204 | 2.735 | **0.034** |
|  | log2(cold time) | 0.058 | 0.234 | 0.249 | 0.812 |
|  | Age | 0.035 | 0.024 | 1.438 | 0.201 |
| Temporal lobe cortex | log2(warm time) | 0.547 | 0.331 | 1.654 | 0.149 |
|  | log2(cold time) | 0.162 | 0.379 | 0.428 | 0.684 |
|  | Age | 0.036 | 0.040 | 0.896 | 0.405 |
| Occipital lobe cortex | log2(warm time) | 0.452 | 0.254 | 1.784 | 0.125 |
|  | log2(cold time) | 0.226 | 0.291 | 0.779 | 0.466 |
|  | Age | -0.001 | 0.030 | -0.024 | 0.982 |
| Frontal lobe white matter | log2(warm time) | 0.573 | 0.231 | 2.480 | **0.048** |
|  | log2(cold time) | -0.009 | 0.265 | -0.036 | 0.973 |
|  | Age | 0.019 | 0.028 | 0.671 | 0.527 |
| Parietal lobe white matter | log2(warm time) | 0.586 | 0.301 | 1.947 | 0.099 |
|  | log2(cold time) | 0.170 | 0.345 | 0.492 | 0.640 |
|  | Age | 0.021 | 0.036 | 0.583 | 0.581 |
| Temporal lobe white matter | log2(warm time) | 0.603 | 0.209 | 2.880 | **0.028** |
|  | log2(cold time) | 0.088 | 0.240 | 0.367 | 0.726 |
|  | Age | 0.017 | 0.025 | 0.667 | 0.530 |
| Occipital lobe white matter | log2(warm time) | 0.585 | 0.194 | 3.008 | **0.024** |
|  | log2(cold time) | 0.084 | 0.223 | 0.375 | 0.720 |
|  | Age | 0.022 | 0.023 | 0.938 | 0.385 |
| Caudate nucleus | log2(warm time) | 0.431 | 0.147 | 2.935 | **0.026** |
|  | log2(cold time) | 0.031 | 0.168 | 0.185 | 0.860 |
|  | Age | -0.016 | 0.018 | -0.923 | 0.392 |
| Internal capsule | log2(warm time) | 0.929 | 0.267 | 3.486 | **0.013** |
|  | log2(cold time) | -0.542 | 0.306 | -1.773 | 0.127 |
|  | Age | 0.002 | 0.032 | 0.052 | 0.960 |
| Hippocampus | log2(warm time) | 0.011 | 0.187 | 0.057 | 0.957 |
|  | log2(cold time) | 0.091 | 0.200 | 0.455 | 0.673 |
|  | Age | 0.018 | 0.023 | 0.794 | 0.472 |
| Thalamus | log2(warm time) | 0.306 | 0.411 | 0.743 | 0.485 |
|  | log2(cold time) | 0.004 | 0.471 | 0.009 | 0.993 |
|  | Age | 0.017 | 0.049 | 0.347 | 0.740 |
| Mesencephalon | log2(warm time) | 0.647 | 0.354 | 1.830 | 0.117 |
|  | log2(cold time) | 0.120 | 0.405 | 0.296 | 0.778 |
|  | Age | 0.097 | 0.042 | 2.297 | 0.061 |
| Pons | log2(warm time) | 0.723 | 0.272 | 2.659 | **0.038** |
|  | log2(cold time) | -0.147 | 0.311 | -0.472 | 0.653 |
|  | Age | 0.064 | 0.033 | 1.963 | 0.097 |
| Medulla oblongata | log2(warm time) | 0.565 | 0.231 | 2.442 | 0.050 |
|  | log2(cold time) | 0.068 | 0.265 | 0.256 | 0.807 |
|  | Age | 0.044 | 0.028 | 1.572 | 0.167 |
| Cervical spinal cord | log2(warm time) | 0.417 | 0.176 | 2.373 | 0.055 |
|  | log2(cold time) | 0.184 | 0.201 | 0.915 | 0.395 |
|  | Age | 0.058 | 0.021 | 2.768 | **0.033** |
| Cerebellum | log2(warm time) | 0.916 | 0.207 | 4.425 | **0.004** |
|  | log2(cold time) | -0.268 | 0.237 | -1.129 | 0.302 |
|  | Age | 0.031 | 0.025 | 1.246 | 0.259 |

**Table S3.** NFL, multiple linear regression results.

| **Brain section** | **Variable** | **Estimate** | **Std. Error** | **t value** | **Pr(>\|t\|)** |
| --- | --- | --- | --- | --- | --- |
| Frontal lobe cortex | log2(warm time) | 0.320 | 0.254 | 1.262 | 0.254 |
|  | log2(cold time) | -0.189 | 0.291 | -0.651 | 0.539 |
|  | Age | -0.029 | 0.030 | -0.945 | 0.381 |
| Parietal lobe cortex | log2(warm time) | 0.510 | 0.210 | 2.427 | 0.051 |
|  | log2(cold time) | -0.298 | 0.241 | -1.236 | 0.263 |
|  | Age | -0.026 | 0.025 | -1.034 | 0.341 |
| Temporal lobe cortex | log2(warm time) | 0.290 | 0.261 | 1.111 | 0.309 |
|  | log2(cold time) | -0.112 | 0.299 | -0.373 | 0.722 |
|  | Age | -0.005 | 0.031 | -0.171 | 0.870 |
| Occipital lobe cortex | log2(warm time) | 0.493 | 0.285 | 1.731 | 0.134 |
|  | log2(cold time) | -0.228 | 0.326 | -0.700 | 0.510 |
|  | Age | -0.022 | 0.034 | -0.649 | 0.540 |
| Frontal lobe white matter | log2(warm time) | 1.557 | 0.462 | 3.371 | **0.015** |
|  | log2(cold time) | -0.796 | 0.529 | -1.504 | 0.183 |
|  | Age | 0.012 | 0.055 | 0.209 | 0.841 |
| Parietal lobe white matter | log2(warm time) | 1.469 | 0.399 | 3.677 | **0.010** |
|  | log2(cold time) | -0.729 | 0.458 | -1.592 | 0.163 |
|  | Age | 0.024 | 0.048 | 0.510 | 0.628 |
| Temporal lobe white matter | log2(warm time) | 1.183 | 0.431 | 2.745 | **0.034** |
|  | log2(cold time) | -0.821 | 0.494 | -1.663 | 0.147 |
|  | Age | -0.015 | 0.052 | -0.282 | 0.787 |
| Occipital lobe white matter | log2(warm time) | 1.187 | 0.422 | 2.810 | **0.031** |
|  | log2(cold time) | -0.735 | 0.484 | -1.517 | 0.180 |
|  | Age | -0.006 | 0.051 | -0.118 | 0.910 |
| Caudate nucleus | log2(warm time) | 0.886 | 0.424 | 2.089 | 0.082 |
|  | log2(cold time) | -0.749 | 0.486 | -1.541 | 0.174 |
|  | Age | 0.005 | 0.051 | 0.099 | 0.924 |
| Internal capsule | log2(warm time) | 0.960 | 0.544 | 1.765 | 0.128 |
|  | log2(cold time) | -1.210 | 0.623 | -1.941 | 0.100 |
|  | Age | -0.003 | 0.065 | -0.042 | 0.968 |
| Hippocampus | log2(warm time) | 0.404 | 0.714 | 0.567 | 0.601 |
|  | log2(cold time) | -0.806 | 0.763 | -1.057 | 0.350 |
|  | Age | -0.043 | 0.088 | -0.493 | 0.648 |
| Thalamus | log2(warm time) | 0.811 | 0.525 | 1.545 | 0.173 |
|  | log2(cold time) | -0.548 | 0.601 | -0.910 | 0.398 |
|  | Age | -0.001 | 0.063 | -0.012 | 0.991 |
| Mesencephalon | log2(warm time) | 1.130 | 0.382 | 2.956 | **0.025** |
|  | log2(cold time) | -0.668 | 0.438 | -1.525 | 0.178 |
|  | Age | -0.004 | 0.046 | -0.077 | 0.941 |
| Pons | log2(warm time) | 1.015 | 0.276 | 3.679 | **0.010** |
|  | log2(cold time) | -0.487 | 0.316 | -1.539 | 0.175 |
|  | Age | -0.002 | 0.033 | -0.067 | 0.948 |
| Medulla oblongata | log2(warm time) | 1.027 | 0.318 | 3.225 | **0.018** |
|  | log2(cold time) | -0.359 | 0.365 | -0.984 | 0.363 |
|  | Age | -0.016 | 0.038 | -0.411 | 0.695 |
| Cervical spinal cord | log2(warm time) | 1.043 | 0.313 | 3.337 | **0.016** |
|  | log2(cold time) | -0.376 | 0.358 | -1.048 | 0.335 |
|  | Age | 0.030 | 0.037 | 0.814 | 0.447 |
| Cerebellum | log2(warm time) | 0.415 | 0.335 | 1.238 | 0.262 |
|  | log2(cold time) | -0.278 | 0.384 | -0.725 | 0.496 |
|  | Age | 0.025 | 0.040 | 0.629 | 0.552 |

**Table S4.** TAU, multiple linear regression results.

| **Brain section** | **Variable** | **Estimate** | **Std. Error** | **t value** | **Pr(>\|t\|)** |
| --- | --- | --- | --- | --- | --- |
| Frontal lobe cortex | log2(warm time) | 0.083 | 0.083 | 1.000 | 0.356 |
|  | log2(cold time) | 0.035 | 0.095 | 0.368 | 0.726 |
|  | Age | -0.017 | 0.010 | -1.697 | 0.141 |
| Parietal lobe cortex | log2(warm time) | 0.055 | 0.125 | 0.442 | 0.674 |
|  | log2(cold time) | -0.002 | 0.143 | -0.011 | 0.992 |
|  | Age | -0.038 | 0.015 | -2.530 | **0.045** |
| Temporal lobe cortex | log2(warm time) | 0.140 | 0.087 | 1.616 | 0.157 |
|  | log2(cold time) | -0.086 | 0.099 | -0.869 | 0.418 |
|  | Age | -0.006 | 0.010 | -0.598 | 0.571 |
| Occipital lobe cortex | log2(warm time) | 0.097 | 0.149 | 0.650 | 0.540 |
|  | log2(cold time) | -0.012 | 0.170 | -0.072 | 0.945 |
|  | Age | -0.030 | 0.018 | -1.668 | 0.146 |
| Frontal lobe white matter | log2(warm time) | -0.318 | 0.120 | -2.642 | **0.038** |
|  | log2(cold time) | -0.140 | 0.138 | -1.012 | 0.350 |
|  | Age | -0.044 | 0.014 | -3.074 | **0.022** |
| Parietal lobe white matter | log2(warm time) | -0.322 | 0.131 | -2.462 | **0.049** |
|  | log2(cold time) | -0.092 | 0.150 | -0.611 | 0.564 |
|  | Age | -0.030 | 0.016 | -1.905 | 0.105 |
| Temporal lobe white matter | log2(warm time) | -0.179 | 0.222 | -0.807 | 0.451 |
|  | log2(cold time) | 0.095 | 0.255 | 0.373 | 0.722 |
|  | Age | -0.042 | 0.027 | -1.573 | 0.167 |
| Occipital lobe white matter | log2(warm time) | -0.283 | 0.163 | -1.736 | 0.133 |
|  | log2(cold time) | -0.026 | 0.187 | -0.140 | 0.893 |
|  | Age | -0.014 | 0.020 | -0.743 | 0.486 |
| Caudate nucleus | log2(warm time) | 0.162 | 0.051 | 3.186 | **0.019** |
|  | log2(cold time) | 0.026 | 0.058 | 0.449 | 0.670 |
|  | Age | 0.003 | 0.006 | 0.504 | 0.632 |
| Internal capsule | log2(warm time) | -0.302 | 0.233 | -1.297 | 0.242 |
|  | log2(cold time) | 0.207 | 0.267 | 0.776 | 0.467 |
|  | Age | 0.005 | 0.028 | 0.187 | 0.858 |
| Hippocampus | log2(warm time) | 0.001 | 0.223 | 0.006 | 0.995 |
|  | log2(cold time) | 0.270 | 0.238 | 1.133 | 0.320 |
|  | Age | -0.020 | 0.028 | -0.714 | 0.515 |
| Thalamus | log2(warm time) | 0.007 | 0.223 | 0.031 | 0.976 |
|  | log2(cold time) | 0.139 | 0.255 | 0.545 | 0.605 |
|  | Age | -0.003 | 0.027 | -0.122 | 0.907 |
| Mesencephalon | log2(warm time) | -0.200 | 0.248 | -0.808 | 0.450 |
|  | log2(cold time) | 0.117 | 0.284 | 0.412 | 0.694 |
|  | Age | 0.012 | 0.030 | 0.388 | 0.712 |
| Pons | log2(warm time) | 0.141 | 0.167 | 0.841 | 0.433 |
|  | log2(cold time) | -0.002 | 0.192 | -0.011 | 0.992 |
|  | Age | -0.012 | 0.020 | -0.623 | 0.556 |
| Medulla oblongata | log2(warm time) | -0.039 | 0.050 | -0.776 | 0.467 |
|  | log2(cold time) | -0.046 | 0.057 | -0.798 | 0.456 |
|  | Age | -0.012 | 0.006 | -1.978 | 0.095 |
| Cervical spinal cord | log2(warm time) | -0.236 | 0.152 | -1.546 | 0.173 |
|  | log2(cold time) | 0.274 | 0.175 | 1.571 | 0.167 |
|  | Age | -0.036 | 0.018 | -1.998 | 0.093 |
| Cerebellum | log2(warm time) | -0.100 | 0.188 | -0.532 | 0.614 |
|  | log2(cold time) | -0.042 | 0.216 | -0.196 | 0.851 |
|  | Age | -0.004 | 0.023 | -0.173 | 0.868 |

**Table S5.** UCHL1, multiple linear regression results.

| **Brain section** | **Variable** | **Estimate** | **Std. Error** | **t value** | **Pr(>\|t\|)** |
| --- | --- | --- | --- | --- | --- |
| Frontal lobe cortex | log2(warm time) | 0.218 | 0.283 | 0.770 | 0.471 |
|  | log2(cold time) | -0.335 | 0.325 | -1.033 | 0.341 |
|  | Age | 0.011 | 0.034 | 0.326 | 0.756 |
| Parietal lobe cortex | log2(warm time) | 0.212 | 0.305 | 0.693 | 0.514 |
|  | log2(cold time) | -0.174 | 0.350 | -0.496 | 0.637 |
|  | Age | 0.007 | 0.037 | 0.190 | 0.855 |
| Temporal lobe cortex | log2(warm time) | 0.287 | 0.390 | 0.737 | 0.489 |
|  | log2(cold time) | -0.343 | 0.447 | -0.767 | 0.472 |
|  | Age | 0.017 | 0.047 | 0.355 | 0.735 |
| Occipital lobe cortex | log2(warm time) | 0.416 | 0.399 | 1.042 | 0.337 |
|  | log2(cold time) | -0.337 | 0.458 | -0.738 | 0.489 |
|  | Age | 0.017 | 0.048 | 0.360 | 0.731 |
| Frontal lobe white matter | log2(warm time) | -0.200 | 0.110 | -1.812 | 0.120 |
|  | log2(cold time) | -0.007 | 0.126 | -0.057 | 0.956 |
|  | Age | -0.010 | 0.013 | -0.745 | 0.484 |
| Parietal lobe white matter | log2(warm time) | -0.118 | 0.119 | -0.986 | 0.362 |
|  | log2(cold time) | -0.006 | 0.137 | -0.041 | 0.969 |
|  | Age | 0.003 | 0.014 | 0.218 | 0.835 |
| Temporal lobe white matter | log2(warm time) | -0.018 | 0.091 | -0.195 | 0.852 |
|  | log2(cold time) | -0.002 | 0.105 | -0.015 | 0.988 |
|  | Age | 0.004 | 0.011 | 0.410 | 0.696 |
| Occipital lobe white matter | log2(warm time) | -0.085 | 0.142 | -0.600 | 0.570 |
|  | log2(cold time) | -0.044 | 0.162 | -0.273 | 0.794 |
|  | Age | 0.012 | 0.017 | 0.695 | 0.513 |
| Caudate nucleus | log2(warm time) | 0.990 | 0.527 | 1.879 | 0.109 |
|  | log2(cold time) | -0.951 | 0.604 | -1.574 | 0.167 |
|  | Age | 0.046 | 0.063 | 0.724 | 0.496 |
| Internal capsule | log2(warm time) | 0.170 | 0.406 | 0.419 | 0.690 |
|  | log2(cold time) | -0.269 | 0.465 | -0.580 | 0.583 |
|  | Age | 0.024 | 0.049 | 0.489 | 0.642 |
| Hippocampus | log2(warm time) | -0.019 | 0.442 | -0.043 | 0.968 |
|  | log2(cold time) | -0.108 | 0.472 | -0.228 | 0.830 |
|  | Age | 0.007 | 0.055 | 0.124 | 0.907 |
| Thalamus | log2(warm time) | 0.522 | 0.545 | 0.958 | 0.375 |
|  | log2(cold time) | -0.824 | 0.624 | -1.320 | 0.235 |
|  | Age | 0.027 | 0.065 | 0.410 | 0.696 |
| Mesencephalon | log2(warm time) | 0.061 | 0.275 | 0.221 | 0.833 |
|  | log2(cold time) | -0.116 | 0.315 | -0.367 | 0.726 |
|  | Age | 0.027 | 0.033 | 0.828 | 0.439 |
| Pons | log2(warm time) | 0.141 | 0.195 | 0.721 | 0.498 |
|  | log2(cold time) | -0.321 | 0.224 | -1.437 | 0.201 |
|  | Age | -0.029 | 0.023 | -1.260 | 0.254 |
| Medulla oblongata | log2(warm time) | 0.134 | 0.208 | 0.645 | 0.543 |
|  | log2(cold time) | -0.207 | 0.238 | -0.869 | 0.418 |
|  | Age | 0.000 | 0.025 | 0.000 | 1.000 |
| Cervical spinal cord | log2(warm time) | -0.032 | 0.118 | -0.274 | 0.793 |
|  | log2(cold time) | -0.012 | 0.135 | -0.089 | 0.932 |
|  | Age | -0.010 | 0.014 | -0.684 | 0.519 |
| Cerebellum | log2(warm time) | 0.214 | 0.336 | 0.635 | 0.549 |
|  | log2(cold time) | -0.303 | 0.386 | -0.786 | 0.462 |
|  | Age | 0.016 | 0.040 | 0.408 | 0.697 |

**Table S6.** Total protein, multiple linear regression results.

| **Brain section** | **Variable** | **Estimate** | **Std. Error** | **t value** | **Pr(>\|t\|)** |
| --- | --- | --- | --- | --- | --- |
| Frontal lobe cortex | log2(warm time) | -0.040 | 0.062 | -0.642 | 0.545 |
|  | log2(cold time) | -0.020 | 0.071 | -0.289 | 0.782 |
|  | Age | -0.005 | 0.007 | -0.619 | 0.559 |
| Parietal lobe cortex | log2(warm time) | -0.003 | 0.062 | -0.043 | 0.967 |
|  | log2(cold time) | -0.023 | 0.071 | -0.320 | 0.760 |
|  | Age | -0.014 | 0.007 | -1.852 | 0.113 |
| Temporal lobe cortex | log2(warm time) | 0.014 | 0.051 | 0.275 | 0.793 |
|  | log2(cold time) | -0.013 | 0.058 | -0.217 | 0.836 |
|  | Age | -0.003 | 0.006 | -0.520 | 0.622 |
| Occipital lobe cortex | log2(warm time) | 0.081 | 0.039 | 2.050 | 0.086 |
|  | log2(cold time) | -0.066 | 0.045 | -1.466 | 0.193 |
|  | Age | -0.001 | 0.005 | -0.314 | 0.764 |
| Frontal lobe white matter | log2(warm time) | 0.105 | 0.072 | 1.455 | 0.205 |
|  | log2(cold time) | -0.151 | 0.073 | -2.076 | 0.093 |
|  | Age | 0.016 | 0.012 | 1.306 | 0.248 |
| Parietal lobe white matter | log2(warm time) | 0.117 | 0.081 | 1.440 | 0.200 |
|  | log2(cold time) | -0.145 | 0.093 | -1.559 | 0.170 |
|  | Age | 0.006 | 0.010 | 0.639 | 0.546 |
| Temporal lobe white matter | log2(warm time) | 0.138 | 0.060 | 2.315 | 0.060 |
|  | log2(cold time) | -0.141 | 0.068 | -2.066 | 0.084 |
|  | Age | 0.009 | 0.007 | 1.251 | 0.258 |
| Occipital lobe white matter | log2(warm time) | 0.125 | 0.074 | 1.683 | 0.143 |
|  | log2(cold time) | -0.139 | 0.085 | -1.634 | 0.153 |
|  | Age | 0.010 | 0.009 | 1.166 | 0.288 |
| Caudate nucleus | log2(warm time) | 0.126 | 0.068 | 1.852 | 0.114 |
|  | log2(cold time) | -0.089 | 0.078 | -1.143 | 0.297 |
|  | Age | 0.014 | 0.008 | 1.678 | 0.144 |
| Internal capsule | log2(warm time) | 0.061 | 0.095 | 0.647 | 0.541 |
|  | log2(cold time) | -0.055 | 0.109 | -0.508 | 0.630 |
|  | Age | 0.009 | 0.011 | 0.827 | 0.440 |
| Hippocampus | log2(warm time) | 0.046 | 0.066 | 0.688 | 0.529 |
|  | log2(cold time) | -0.051 | 0.071 | -0.715 | 0.514 |
|  | Age | 0.010 | 0.008 | 1.261 | 0.276 |
| Thalamus | log2(warm time) | 0.160 | 0.087 | 1.836 | 0.116 |
|  | log2(cold time) | -0.135 | 0.100 | -1.352 | 0.225 |
|  | Age | 0.016 | 0.010 | 1.480 | 0.189 |
| Mesencephalon | log2(warm time) | 0.074 | 0.113 | 0.658 | 0.535 |
|  | log2(cold time) | -0.133 | 0.129 | -1.031 | 0.343 |
|  | Age | 0.022 | 0.014 | 1.634 | 0.153 |
| Pons | log2(warm time) | 0.125 | 0.074 | 1.695 | 0.141 |
|  | log2(cold time) | -0.131 | 0.085 | -1.551 | 0.172 |
|  | Age | 0.010 | 0.009 | 1.076 | 0.323 |
| Medulla oblongata | log2(warm time) | 0.174 | 0.076 | 2.303 | 0.061 |
|  | log2(cold time) | -0.174 | 0.087 | -2.011 | 0.091 |
|  | Age | 0.013 | 0.009 | 1.440 | 0.200 |
| Cervical spinal cord | log2(warm time) | 0.090 | 0.056 | 1.599 | 0.161 |
|  | log2(cold time) | -0.086 | 0.064 | -1.342 | 0.228 |
|  | Age | 0.013 | 0.007 | 1.963 | 0.097 |
| Cerebellum | log2(warm time) | 0.030 | 0.062 | 0.481 | 0.648 |
|  | log2(cold time) | -0.011 | 0.071 | -0.152 | 0.884 |
|  | Age | 0.002 | 0.007 | 0.332 | 0.751 |

**Figure S1.** Histograms of P-values from multiple linear regression analyses. All studied proteins.

# 3. Concentration diagrams

**Figure S2.** Concentrations of GFAP, MBP, NFL, Tau, UCHL1 and Total protein in selected anatomical regions of the CNS, presented in diagrams.

# 4. Association between warm time and concentration of MBP and NFL

**Figure S3.** Association between warm time and MBP in all CNS regions. Increased duration of warm time was associated with higher concentrations of MBP in all CNS regions except hippocampus.

**Figure S4.** Correlation between warm time and NFL in all CNS regions. Increased duration of warm time was associated with higher concentrations of NFL in all CNS regions except hippocampus.

# 5. Validation data

A dilution linearity assessment was performed for two of the assays, the GFAP (Human GFAP DuoSet ELISA-kit, R&D Systems™. Catalog no.: DY2594-05), and UCHL1 (Human UCH-L1/PGP9.5 DuoSet ELISA, R&D Systems™. Catalog no.: DY6007-05). For GFAP three samples were tested from one of the donors; frontal cortex, frontal white matter and internal capsule (Fig. S5-S7) and for UCHL1 two samples were tested; frontal cortex and internal capsule (Fig. S8-S9). A spike and recovery test was performed for UCHL1 (Table S7).

**Figure S5.** Dilution linearity of GFAP assay in frontal cortex. DF = dilution factor, and * = out of range of standard curve.

**Figure S6.** Dilution linearity of GFAP assay in frontal white matter. DF = dilution factor, and * = out of range of standard curve.

**Figure S7.** Dilution linearity of GFAP assay in internal capsule. DF = dilution factor, and * = out of range of standard curve.

**Figure S8.** Dilution linearity of UCHL1 assay in frontal cortex. DF = dilution factor, and * = out of range of standard curve.

**Figure S9.** Dilution linearity of UCHL1 assay in internal capsule. DF = dilution factor, and * = out of range of standard curve.

**Table S7.** Spike and recovery test for UCHL1. The test was performed on two samples, frontal cortex and internal capsule, at 10,000 times dilution. Spikes were 0 (no spike), 0.5 (medium spike), and 1.0 (high spike) ng/ml and added to the samples. Blank = reagent diluent used in the assays to dilute the samples.

|  | **No spike** | **Medium spike** | **NET** | **Recovery** | **High spike** | **NET** | **Recovery** |
| --- | --- | --- | --- | --- | --- | --- | --- |
| **Frontal cortex** | 0.442 | 0.738 | 0.296 | 99% | 1.252 | 0.810 | 120% |
| **Internal capsule** | 0.315 | 0.579 | 0.264 | 89% | 0.957 | 0.642 | 95% |
| **Blank** | 0.000 | 0.298 | 0.298 | 100% | 0.675 | 0.675 | 100% |
